# Comparative transcriptome profiling analysis provides insight into the mechanisms for sugar change in Chinese jujube (*Ziziphus jujuba* Mill.) under rain‐proof cultivation

**DOI:** 10.1002/tpg2.20341

**Published:** 2023-05-05

**Authors:** Qing Ji, Ruifang Wang, Kai Chen, Zhengwan Xie, Sunyang Li, Dawei Wang, Ao Zhang, Yumei Xu, Shenghui Li, Junjun Cui, Sha Liu, Jun Zhou, Lihu Wang

**Affiliations:** ^1^ Puer University Puer Yunnan China; ^2^ School of Landscape and Ecological Engineering Hebei University of Engineering Handan China; ^3^ College of Life Science and Engineering North Minzu University Yinchuan China; ^4^ Key Laboratory for Forest Resource Conservation and Utilization in the Southwest Mountains of China, Ministry of Education Southwest Forestry University Kunming China

## Abstract

Chinese jujube (*Ziziphus jujuba* Mill.) is a globally popular and economically important fruit that is rich in bioactive compounds with strong anti‐cancer effects. Rain‐proof cultivation is widely used to cultivate Chinese jujube, as it helps avoid rainfall damage during fruit harvest. Although the sugar content of jujube fruits differs between rain‐proof and open‐field cultivation, the underlying molecular mechanisms are unknown. Here, we analyzed the levels of sugar content, sugar accumulation pattern, and transcriptome profiles of jujube fruits at five developmental stages grown under rain‐proof and open‐field cultivation modes. The sugar content of jujube fruits was significantly higher under rain‐proof cultivation than under open‐field cultivation, although the sugar composition and sugar accumulation patterns were comparable. Comparative analysis of transcriptomic profiles showed that rain‐proof cultivation enhanced the intrinsic metabolic activity of fruit development. Gene expression and correlation analyses suggested that *Zj*SPS, *Zj*SS, *Zj*HXK, and *Zj*INV regulate the development‐related changes in sugar content in jujube fruits grown under rain‐proof cultivation. Temperature, humidity, and moisture conditions were key climatic factors affecting sugar accumulation. Our results provide insights into the molecular mechanisms regulating sugar content and sugar accumulation in Chinese jujube fruits grown under rain‐proof cultivation, and we provide genetic resources for studying the development mechanism of Chinese jujube fruit.

## Core Ideas

- This study reveals molecular mechanisms for sugar change in Chinese jujube under rainproof cultivation.
- We predicted that ZjSPS, ZjSS, ZjHXK, and ZjINV play an important role in the change of sugar in jujube fruits.
- This study provides abundant genetic resources for the study of sugar accumulation metabolism of Chinese jujube.

## 1 INTRODUCTION

Chinese jujube (*Ziziphus jujuba* Mill.), also known as the Chinese date or red date (Wang et al., 2019a), is the most important fruit crop of the Rhamnaceae family. With a domestication history of 3000 years, it is one of the most widely cultivated and economical fruit trees in China, and has been planted on more than 2 million hectares. The Chinese jujube is widely distributed because of its enjoyable flavor and has been introduced in at least 47 countries (Wang et al., 2019b). Moreover, the jujube fruit has many compounds that benefit human health (Dabaghian et al., 2018; Ji et al., 2020), including vitamin C, sugars, and mineral nutrients (Gao et al., 2013; Ji et al., 2021, 2022).

Fruit flavors are formed though a complicated mechanism. The quality of commercially available jujube mainly depends on its content of biologically active compounds, such as sugars, acids, polyphenols, and flavonoids. The combination of these compounds affects the flavor of jujube fruit, and the levels of these compounds in the fruit are affected by the environmental changes, such as light, water, and temperature (Gao et al., 2011). Rain‐proof cultivation is a simple form of facility cultivation in which plants are sheltered only from the rain and not from the wind. This system regulates the environment for plant growth, thereby improving the crop yield and quality. In the past two decades, rain‐proof cultivation has been increasingly used to cultivate fruit tree including but are not limit to apple, pear, and grape (Gao et al., 2016; Meng et al., 2017).

Jujube naturally ripens in late September and early‐ to mid‐October (Zhang et al., 2020). These months are often rainy and have humid weather with high temperatures especially in southern China, and these environmental factors have a considerable influence on the fruit quality. The rain‐proof cultivation of jujube helps avoid the cracking of fruits due to rain washing and has allowed the yearly expansion of the cultivation area of Chinese jujube. Sugar is the most important compound in jujube fruits, and sugar content is an important factor affecting fruit quality and flavor formation. The accumulation of soluble sugars in fresh fruits directly affects the commercial value of the fruit (Guo et al., 2015; Velasco et al., 2010). Fructose, glucose, and sucrose are considered the most important sugars in Chinese jujube. The quality of the jujube fruit has been improved to varying degrees using rain‐proof cultivation, especially through an increase in the total sugar content. Previous studies on the quality of Chinese jujube fruits grown under rain‐proof cultivation mainly focused on changes in total sugar content (Ji et al., 2018; Yuan et al., 2012). However, changes in the levels of sugar components and the associated molecular mechanisms have not been investigated.

Recent studies have used transcriptome analysis to elucidate the molecular mechanisms underlying disease resistance, insect resistance, trait development, and growth in plants. The genome sequencing of two Chinese jujube cultivars (“Dongzao” and “Junzao”) in 2014 (Liu et al., 2014) and 2016 (Huang et al., 2016a) has provided a foundation for exploring jujube fruit biology. To date, studies have identified hundreds of gene families involved in fruit sugar metabolism (Wu et al., 2014). Additionally, the sweet/acidic taste that determines the flavor of the jujube fruit has also been investigated (Huang et al., 2016b). In short, it is now possible to use transcriptome analysis to reveal the molecular mechanisms underlying change in sugar content in Chinese jujube plants cultivated under rain‐proof (RP) and open‐field (OF) cultivation systems.

In this study, we investigated the sugar composition, sugar contents, and transcriptomic changes in jujube fruits during fruit development in rain‐proof and open‐field cultivation. We used automatic weather stations to generate and analyze the microclimate changes in the growth environment of jujube fruits and their effects on the changes in sugar content under the two cultivation modes, and we combined physiological and transcriptomic data to analyze the key pathways and genes affecting the changes in sugar content in RP and OF cultivations. The results of this study enhance the available transcriptomic resources for the Chinese jujube and are of great significance for the future research on sugar accumulation in this fruit.

**Core Ideas**

- This study reveals molecular mechanisms for sugar change in Chinese jujube under rainproof cultivation.
- We predicted that ZjSPS, ZjSS, ZjHXK, and ZjINV play an important role in the change of sugar in jujube fruits.
- This study provides abundant genetic resources for the study of sugar accumulation metabolism of Chinese jujube.

## 2 MATERIALS AND METHODS

### 2.1 Plant materials and sampling

We used the Chinese jujube cultivar, “Dongzao,” and selected 12‐year‐old trees for this study. The trees were planted in the experimental field (located in Xuefeng ecological park, Yiliang county, Yunnan Province, China) and divided into two groups. In Group 1, 10 trees were grown in an RP cultivation, in which the shelter was a single‐arch shed covered with polyethylene film material and framed by cement columns and steel frames. The width and height of the shed were 600 and 300 cm, respectively. The side skirt of the shed was 30 cm above the ground. In Group 2, 10 trees were grown in an OF cultivation without the polyethylene film. All sample trees were grown and routinely managed in Yiliang county (elevation, 1500 m; annual precipitation, 912.2 mm; annual sunshine time, 2177.3 h) in Yunnan province, China. The soil nutrient status of the experimental garden is shown in Table 1 (Ji et al., 2016).

**TABLE 1:** Soil nutrient status in the experimental orchard (Ji et al., 2016)

Soil depth (cm)	pH value	Organic matter (g·kg^−1^)	Hydrolyzable N (g·kg^−1^)	Available P (g·kg^−1^)	Available Zn (g·kg^−1^)	Available Fe (g·kg^−1^)
0–20	5.52	22.40	130.00	23.10	9.33	80.00
20–40	4.58	15.00	93.50	8.94	6.46	75.20
Soil depth (cm)	Available Mg (g·kg^−1^)	Available Mn (g·kg^−1^)	Available Ca (g·kg^−1^)	Available Cu (g·kg^−1^)	Available Na (g·kg^−1^)	Available K (g·kg^−1^)
0–20	17.70	10.50	468.00	3.62	9.41	257.00
20–40	13.20	11.40	406.00	3.56	10.40	115.00

Fruits from five different developmental stages were harvested at 25 (young stage; Y), 39 (enlargement stage; EN), 74(white mature stage; WM), 83 (half‐red stage; HR), and 99 (full‐red stage; FR) days after anthesis. The jujube fruits were collected at the same time of day, snap‐frozen in liquid nitrogen, and stored at −80°C until further processing. Three biological repeats were performed for each stage, and each biological replicate was a mixture of three to five fruits.

### 2.2 Acquisition of automatic weather station data

Two automatic weather stations (NHQXZ601) (Nenghui Technology, Wuhan, China) were placed in the RP cultivation and OF cultivation environment. The weather station automatically recorded the atmospheric temperature, atmospheric humidity, wind speed, soil temperature, soil humidity, rainfall, illuminance, ultraviolet radiation level, and photosynthesis in the two cultivation modes every 10 min.

### 2.3 Sugar content determination

The levels of different sugar components (fructose, glucose, and sucrose) were measured via high‐performance liquid chromatography (HPLC) (Hitachi L‐2000, Japan) following previously published methods (Gao et al., 2012) with slight modifications. The HPLC standards included three solutions: fructose (3 mg/mL), sucrose (6 mg/mL), and glucose (6 mg/mL). A total of 1.0 g of jujube powder was accurately weighed and passed through an 80‐mesh sieve, 40 mL of 80% ethanol was added, and ultrasonic extraction was performed at 40°C for 15 min. The solution was cooled and centrifuged at 4°C for 15 min, and the supernatant was transferred to a conical flask. To fully extract the sugar, the residue was extracted two more times, and 30 mL of 80% ethanol was added each time. The supernatant was combined and evaporated to dryness at 45°C using a rotary evaporator. The volume was adjusted to 10 mL with ultrapure water. After filtration through a 0.22 μM aqueous membrane, the extract was used for HPLC analysis. The mobile phase of HPLC was a mixture of acetonitrile and water (80:20) at a flow rate of 1 mL/min. Every experiment was conducted with three independent technical replicates.

### 2.4 RNA extraction, quantification, sequencing and differential gene expression analyses

Total RNA from each fruit sample was isolated using TRIzol reagent (Invitrogen, Carlsbad, CA, USA), and an 2100 Bioanalyzer system (Agilent Technologies, Inc., Santa Clara, CA, USA) was used to detect RNA. Sequencing libraries were constructed with the NEBNext® Ultra™ RNA Library Prep Kit (New England Biolabs, Ipswich, MA, USA) according to the manufacturer's recommendations. The mRNA was enriched with mRNA Capture Beads and fragmented. Single‐stranded cDNA was synthesized using random hexamers, following which the double‐stranded cDNA was synthesized. After end‐repair, the A tail and adapter were added, and the cDNA fragments 150–200 bp in length were selected for sequencing. The sequencing libraries were generated via polymerase chain reaction (PCR), and their quality was verified. The libraries were sequenced using a HiSeq 2000 sequencer (Illumina, San Diego, CA, USA). The adapters and low‐quality reads were removed from the raw data, to obtain the clean data for which the Q20 and Q30 were calculated. The high‐quality clean reads were mapped on the Jujube reference genome (v1.1) using Hierarchical Indexing for Spliced Alignment of Transcripts (HISAT2, version 2.0.5) (Kim et al., 2019) with default parameters. The numbers of uniquely mapping read counts were normalized to fragments per kilobase of million mapped (FPKM) using Cufflinks (v2.0.2) (Trapnell et al., 2012) to obtain the relative levels of expression for each sample.

We performed differential expression analysis using Cufflinks. Genes with adjusted *p*‐values (*q*‐value) ≤0.05, and an absolute value of log_2_ (fold change) ≥1 were categorized as differentially expressed genes (DEGs). These were used to analyze the patterns of gene expression in the jujube fruits grown in RP and OF cultivations.

### 2.5 Gene ontology and Kyoto encyclopedia analysis of DEGs

The DEGs were functionally annotated using Gene Ontology (GO) enrichment analysis (Gene Ontology Consortium, 2004) according to GO:: TermFinder (Boyle et al., 2004), as well as Kyoto Encyclopedia of Genes and Genomes (KEGG) pathway analyses (Kanehisa & Goto, 2000). A corrected *p*‐value < 0.05 was set as the threshold for significantly enriched GO and KEGG terms. The figures were prepared using the OriginPro 8.5 software (OriginLab, Inc.).

### 2.6 Multiple sequence alignment and phylogenetic tree construction of jujube and Arabidopsis proteins

We downloaded 90 protein sequences of *Arabidopsis* from The *Arabidopsis* Information Resource (http://www.Arabidopsis.org/). The full‐length proteins of jujube and *Arabidopsis* were ClustalW‐aligned, and the phylogenetic tree was constructed using the neighbor‐joining method with 1000 bootstrap replicates in MEGA6 (Tamura et al., 2013) with default settings.

### 2.7 Gene co‐expression network construction and hub gene identification

The WGCNA(weighted gene co‐expression network analysis) R package (Langfelder & Horvath, 2008) was used to construct gene co‐expression networks and select highly modules or correlated (or hub) genes that may be strongly associated with sugar biosynthesis. The interaction networks among were drawn using Cytoscape 3.6.1 (Ideker 2011). A *p*‐value < 0.05 and correlation coefficient >0.5 was considered to be a significant correlation between the module and traits.

### 2.8 Quantitative real‐time PCR verification of DEGs

The results of RNA‐Seq were validated via quantitative real‐time PCR (qRT‐PCR). The primers were designed in Primer (v3.0) and are listed in Table S1. Quantitative real‐time PCR was performed using the biological replicate of every fruit sample and three technical replicates. The relative expression level of each gene was calculated by normalization to actin mRNA levels. Changes in expression were calculated using the 2^−△△^Ct method (Livak & Schmittgen, 2001) and the *Zj*Actin gene (107425564) (Liu et al., 2019) was used as an internal reference gene.

### 2.9 Statistical analysis

Data were processed and statistically analyzed in Excel (Microsoft Office, 2018). Following an analysis of variance (ANOVA), we used Student's *t*‐test to evaluate the statistical significance of difference between groups. The UpSet plot for DEGs and the heatmap of gene expression of gene in the sugar metabolism pathway were generated with the TBtools software (Chen et al., 2018). In those plots, the color scales indicate the FPKM counts, and the ratios were log2 transformed. The principal component analysis (PCA) plots, and Boxplot were created using the “ggplot2” package (Wickham, 2011) in R statistical software (R Development, Core Team, 2018). Correlation analysis was performed using Pearson product‐moment correlation in the “corrplot” in R (Wei et al., 2017).

## 3 RESULTS

### 3.1 The microclimate of RP cultivation changed

**FIGURE 1:**
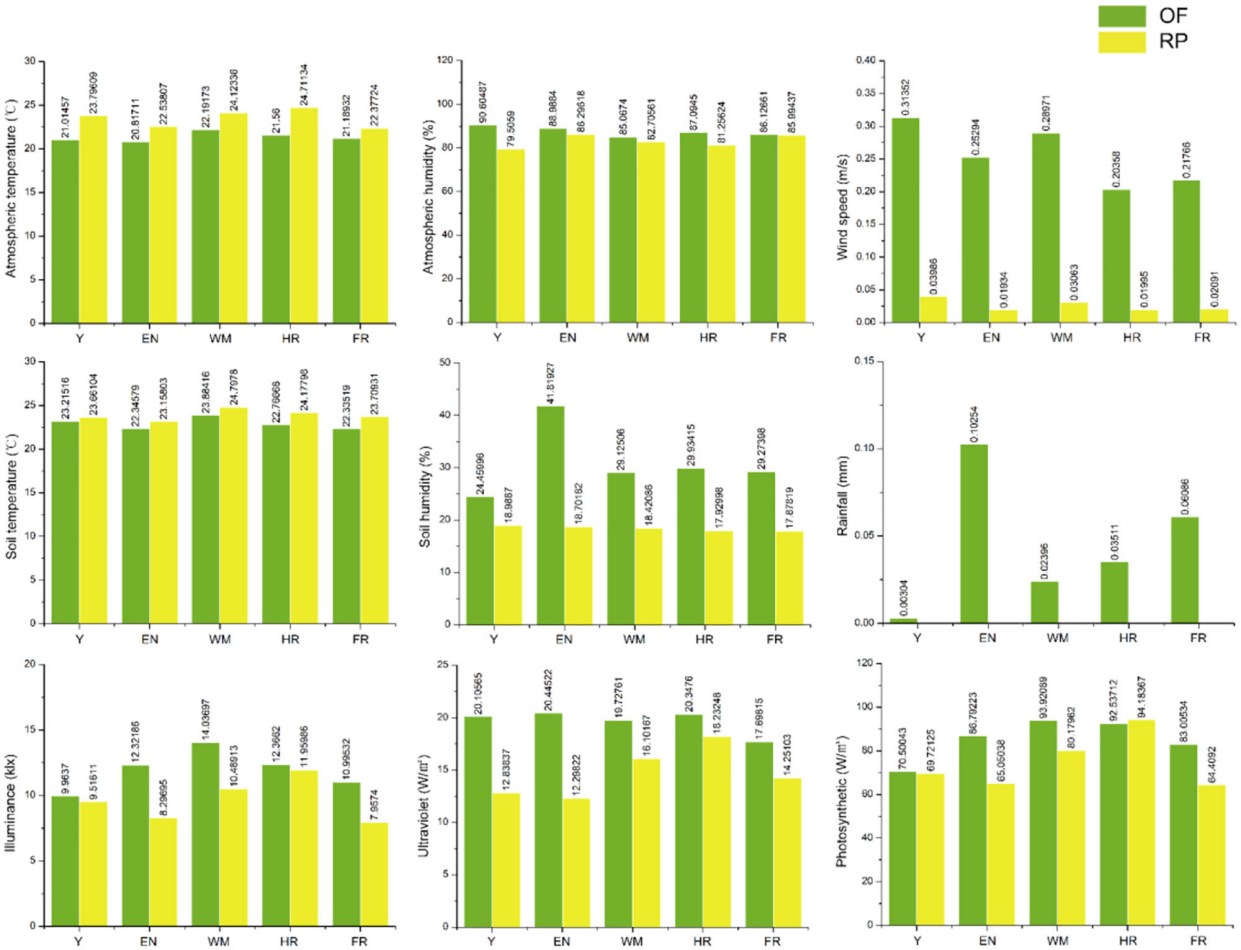
The changes in the environmental factors at different developmental stages of jujube fruit. EN, enlargement stage; FR, full‐red stage; HR, half‐red stage; OF, open‐field; RP, rain‐proof; WM, white mature stage; Y, young stage.

The mean values of meteorological factors for different developmental stages of jujube fruit were analyzed (Figure 1). The atmospheric and soil temperatures in the five developmental stages under RP cultivation were higher than those under OF cultivation, while the humidity of the air and soil had a contrasting trend. The wind speed in the OF cultivation was approximately ten times that in the RP cultivation. Rainfall under RP cultivation was zero. In the OF cultivation, rainfall began to increase after the Y stage and was the highest during the EN stage. RP cultivation reduced the radiation of ultraviolet light on fruits.

### 3.2 Sugar composition and content in jujube fruits grown in RP and OF cultivations

**FIGURE 2:**
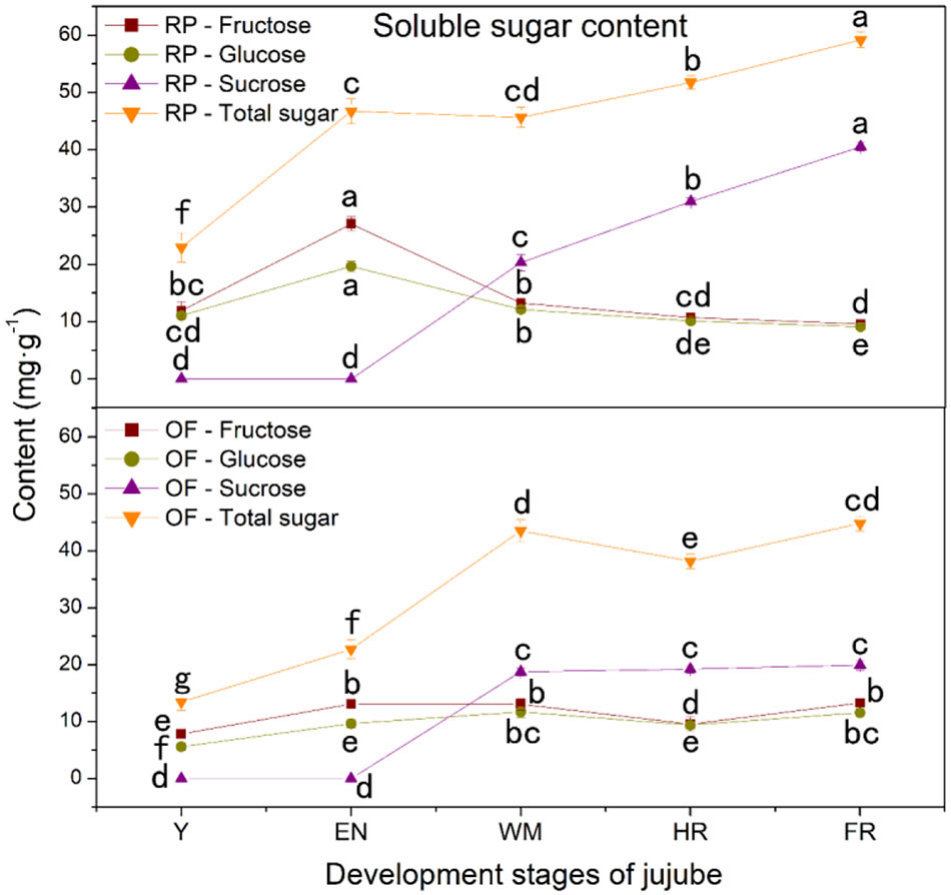
Levels of sucrose, glucose, fructose, and total sugar at various stages in jujube fruit development. The total sugar level is the sum of fructose, glucose, and sucrose levels. Vertical bars show the standard deviations (*n* = 3). EN, enlargement stage; FR, full‐red stage; HR, half‐red stage; OF, open‐field; RP, rain‐proof; WM, white mature stage; Y, young stage.

Sugar is an important indicator of the flavor and nutritional quality of jujube fruit. The accumulation of soluble sugar (including sucrose, glucose, and fructose) directly affects the commercial value of the fruit (Guo et al., 2015; Velasco et al., 2010). In this study, the fruits from the RP cultivation were more flavorful than those from the OF cultivation, although the accumulation of sugar components showed comparable trends in the two cultivation modes (Figure 2). Fructose and glucose were the main components in the early stages (Y and EN) of jujube fruit development, but the sucrose levels were too low for detection. When the jujube reached the WM stage, the sucrose levels increased significantly, and sucrose became the main sugar component of the jujube fruits. In the fruits from the RP cultivation, the levels of fructose and glucose decreased gradually after the EN stage, whereas the levels of sucrose increased significantly. In the FR stage, fruits from the RP cultivation had higher levels of sucrose and total sugar and lower levels of fructose and glucose, compared to fruits from the OF cultivation.

### 3.3 Transcriptome profiles of jujube fruits grown in RP and OF cultivations

We constructed 30 libraries for RNA‐Seq using total RNA isolated from the jujube fruit samples, as described above. The sequence qualities of 15 libraries (RP cultivation), had been verified by a previous research group. The 15 libraries obtained from fruits grown in the OF cultivation generated 48,338,618–50,619,970 raw reads (Table S2). After filtration, a total of 48,271,872–50,544,672 clean reads were obtained from these libraries, with an average Clean Q20 and Clean Q30 of 97.06% and 93.09%, respectively. Thus, the sequencing qualities of fruits grown in RP and OF cultivations were suitable for further analysis. The raw transcriptome sequences of the 30 libraries have been deposited in the NCBI sequence read archive under the accession number SRP162927 (Qing et al., 2019). The detailed information regarding RNA sequencing has been summarized in Table S2.

**FIGURE 3:**
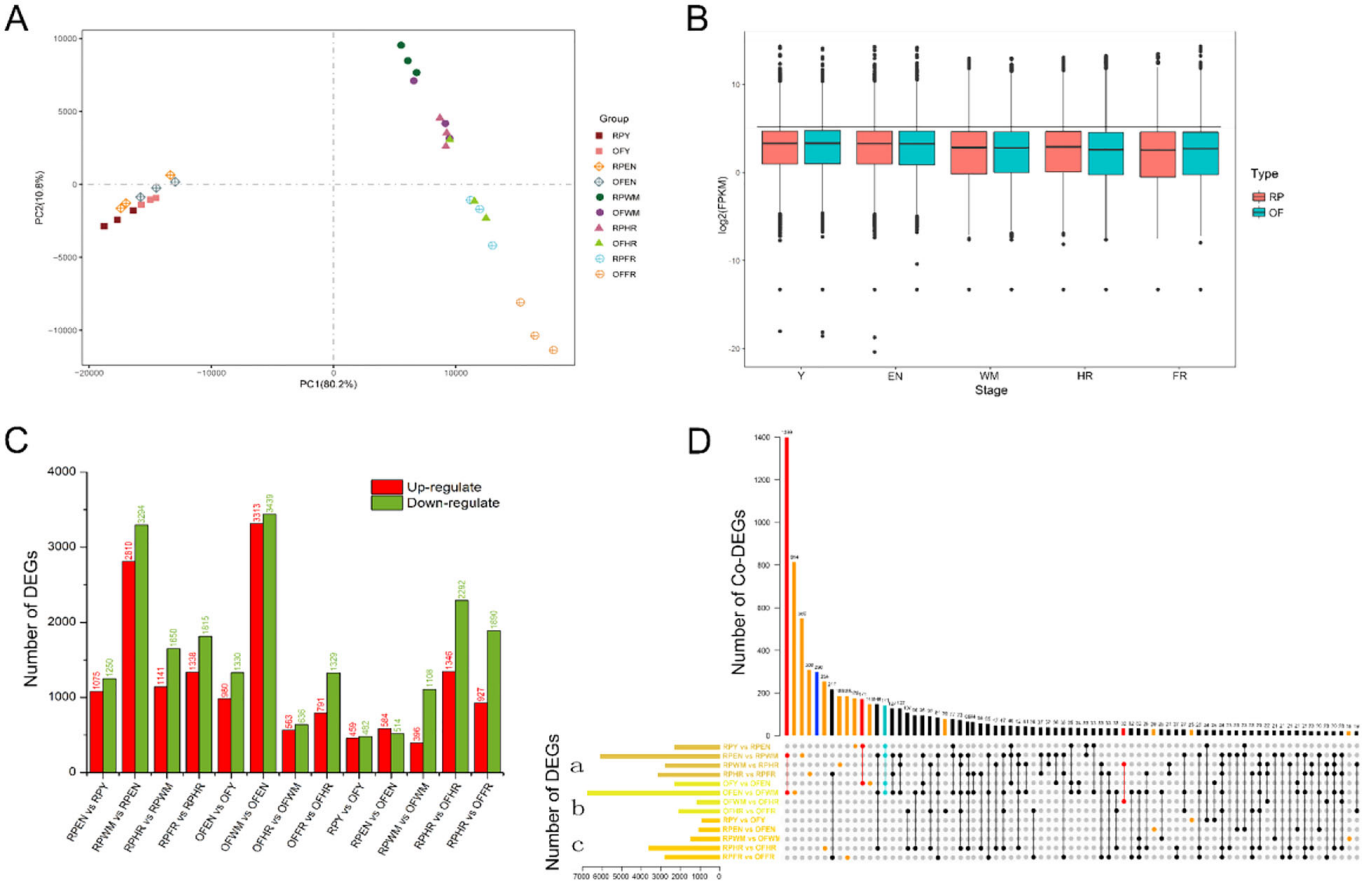
Principal component analysis (PCA) of gene expression among biological replicates of jujube, and the UpSet plot of differentially expressed genes (DEGs). (A) PCA among biological replicates. (B) Boxplots of gene expression in fruits grown in the RP and OF cultivation. (C) Analysis of the number of DEGs between different groups. (D) Venn diagram of DEGs. The left side represents the number of DEGs in different comparison groups, and the right side represents genes that are differentially co‐expressed: (a) Comparison between adjacent periods of fruit development under RP cultivation. (b) Comparison between adjacent periods of fruit development under OF cultivation. (c) Comparison between the same developmental periods in RP and OF cultivation. OF, open‐field; RP, rain‐proof.

To assess the overall quality of the RNA‐Seq data, the FPKM values derived from biological replicates were used for a PCA (Figure 3A). The gene expression patterns were highly consistent between different biological replicates, indicating the high reproducibility of our RNA‐Seq experiment and the high reliability of our sequencing data.

The samples from the Y and EN stages showed a lower collection than samples from the WM, HR, and FR stages. Additionally, the RP samples taken in the last three stages were significantly clustered. The OF samples also showed a similar pattern, indicating that the different developmental stages had different gene expression patterns. Gene expression patterns were also influenced by RP cultivation, as the overall level of gene expression was higher in the RP cultivation group than in the OF cultivation group (Figure 3B). The overall level of gene expression decreased with the maturation of jujube fruits in both cultivation modes (Figure 3B).

### 3.4 DEGs in jujube fruit grown in RP and OF cultivation

We identified a total 20164 genes from all samples using the FPKM method (Table S3), and the DEGs were used for further analysis. We performed comparative analysis of DEGs in four sample pairs (EN vs Y, WM vs EN, HR vs WM, and FR vs HR) under RP and OF cultivations to identify the DEGs during fruit development. In the RP samples, we identified 2325 (1075 upregulated, 1250 downregulated), 6104 (2810, 3294), 2791 (1141, 1650), 3153 (1338, 1815) DEGs in the EN versus Y, WM versus EN, HR versus WM, and FR versus HR comparisons, respectively. In the OF samples, we found 2310 (980 upregulated, 1330 downregulated), 6752 (3313, 3439), 1199 (563, 636), and 2120 (791, 1329) DEGs in EN versus Y, WM versus EN, HR versus WM, and FR versus HR comparisons, respectively (Figure 3C,D; Table S4). Additionally, we performed comparative analysis of DEGs between fruits at the same developmental stage in RP and OF cultivation (RPY vs OFY, RPEN vs OFEN, RPWM vs OFWM, RPHR vs OFHR, and RPFR vs OFFR). We found 941 (459 upregulated, 482 downregulated), 1098 DEGs (584, 514), 1504 (396, 1108), 3638 (1346, 2292), and 2817 DEGs (927, 1890) in RPY versus OFY, RPEN versus OFEN, RPWM versus OFWM, RPHR versus OFHR, and RPFR versus OFFR, comparisons, respectively (Figure 3C,D; Table S4). We also compared the results of adjacent developmental stages among fruits in the same cultivation mode (Figure 3D(a,b)). These results showed in fruits from the two cultivation modes, the number of DEGs showed the same patterns of differences. The number of DEGs in the EN versus WM comparison was the highest in the two cultivation modes and was significantly higher than those in the other comparisons between stages (Figure 3 D(a,b)). The number of DEGs decreased after the WM stage (Figure 3D(a,b)), and the number of downregulated genes showing significant differences was higher than the number of upregulated genes showing significant differences (Figure 3C).

**FIGURE 4:**
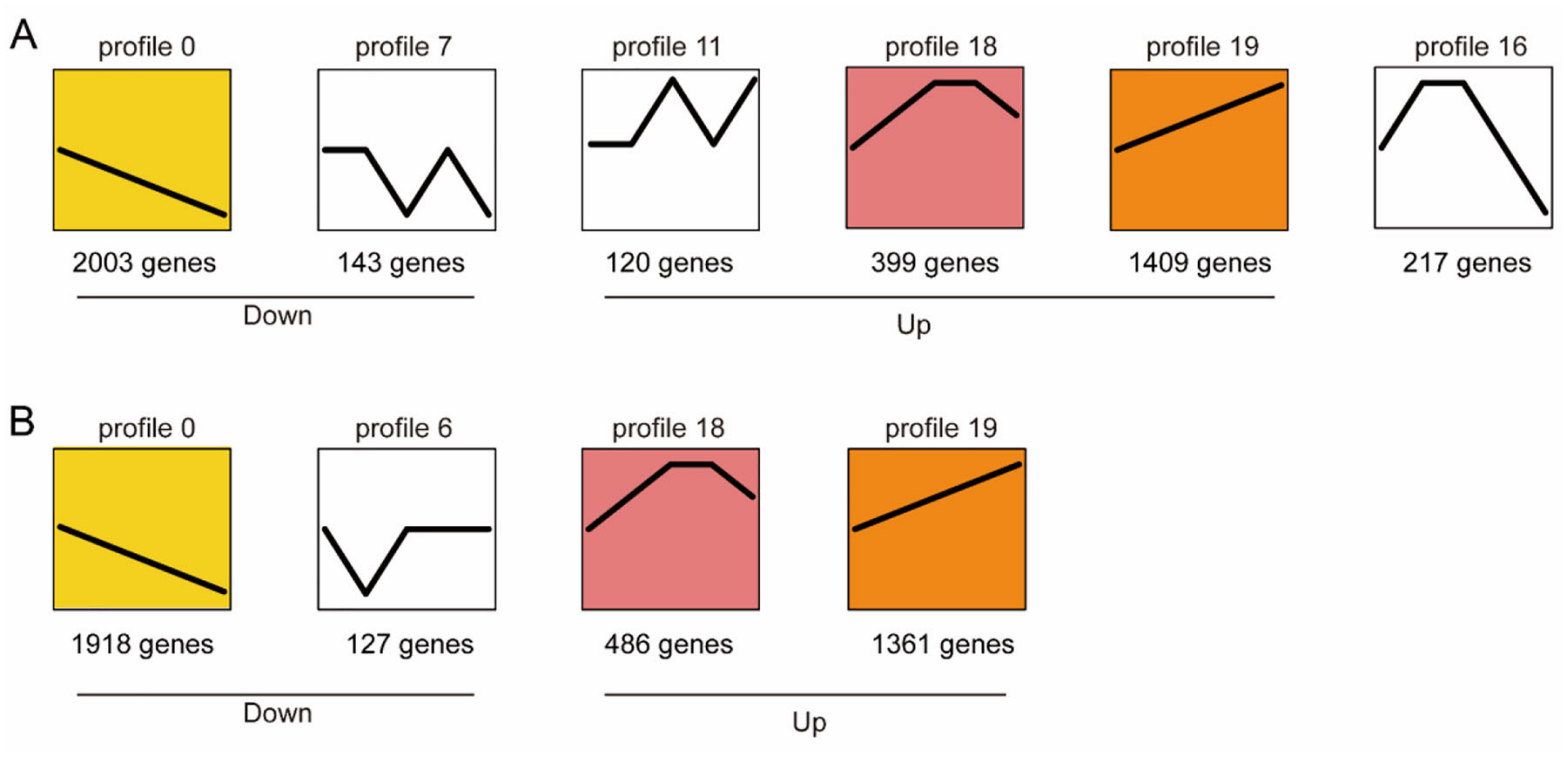
Expression profiles of the identified differentially expressed genes (DEGs). (A) Expression profiles of identified DEGs from the rain‐proof (RP) cultivation. (B) Expression profiles of identified DEGs from the open‐field (OF) cultivation.

To examine the expression profiles of the identified DEGs, 4764 (RP) and 4373(OF) DEGs were clustered into 20 profiles using the Short Time‐Series Expression Miner (STEM) (Ernst & Bar‐Joseph, 2006). In particular, 4291 DEGs from the RP group were clustered into six profiles (*p*‐value < 0.05), including two types of downregulated patterns (Profiles 0 and 7), three upregulated patterns (Profile 11, 18, and 19), and one biphasic expression pattern (Profile 16) (Figure 4A). In the OF group, 3892 DEGs were clustered into four profiles (*p*‐value < 0.05), including two downregulated patterns (Profiles 0 and 6) and two upregulated patterns (Profiles 18 and 19) (Figure 4B).

### 3.5 GO term analysis of DEGs

**FIGURE 5:**
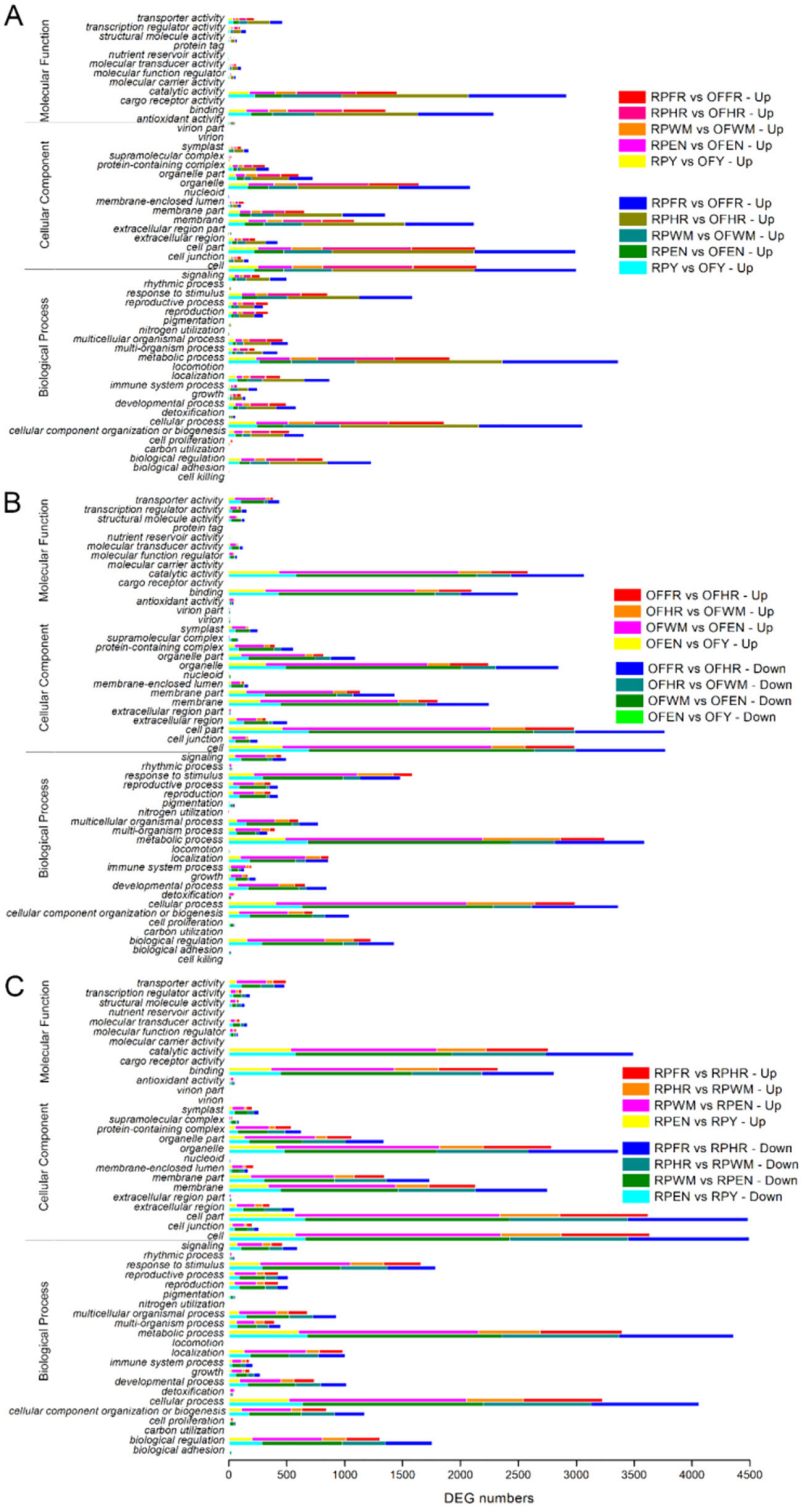
Gene Ontology annotation for differentially expressed genes (DEGs). (A) Comparisons between the same developmental stages under rain‐proof (RP) and open‐field (OF) cultivations. (B) Comparisons between the adjacent stages of fruit development under OF cultivation. (C) Comparisons between the adjacent stages of fruit development under RP cultivation.

The DEG clusters were subsequently used for GO term analysis and were assigned to three core categories: cellular component, biological process, and molecular function. In the three comparison groups, DEGs were mainly enriched in 52 classification entries (Level 2) among the three core categories of GO terms (Figure 5). The most enriched DEGs were involved in catalytic activity and binding in the molecular function category; organelle, membrane part, membrane, cell part and cell in the cell component category; and response to stimulus, metabolic process, cellular process, and biological regulation in the cellular process category. In the majority of the 11 most enriched entries, the number of downregulated genes exceeded the number of upregulated genes.

### 3.6 KEGG pathway enrichment analysis of DEGs

**FIGURE 6:**
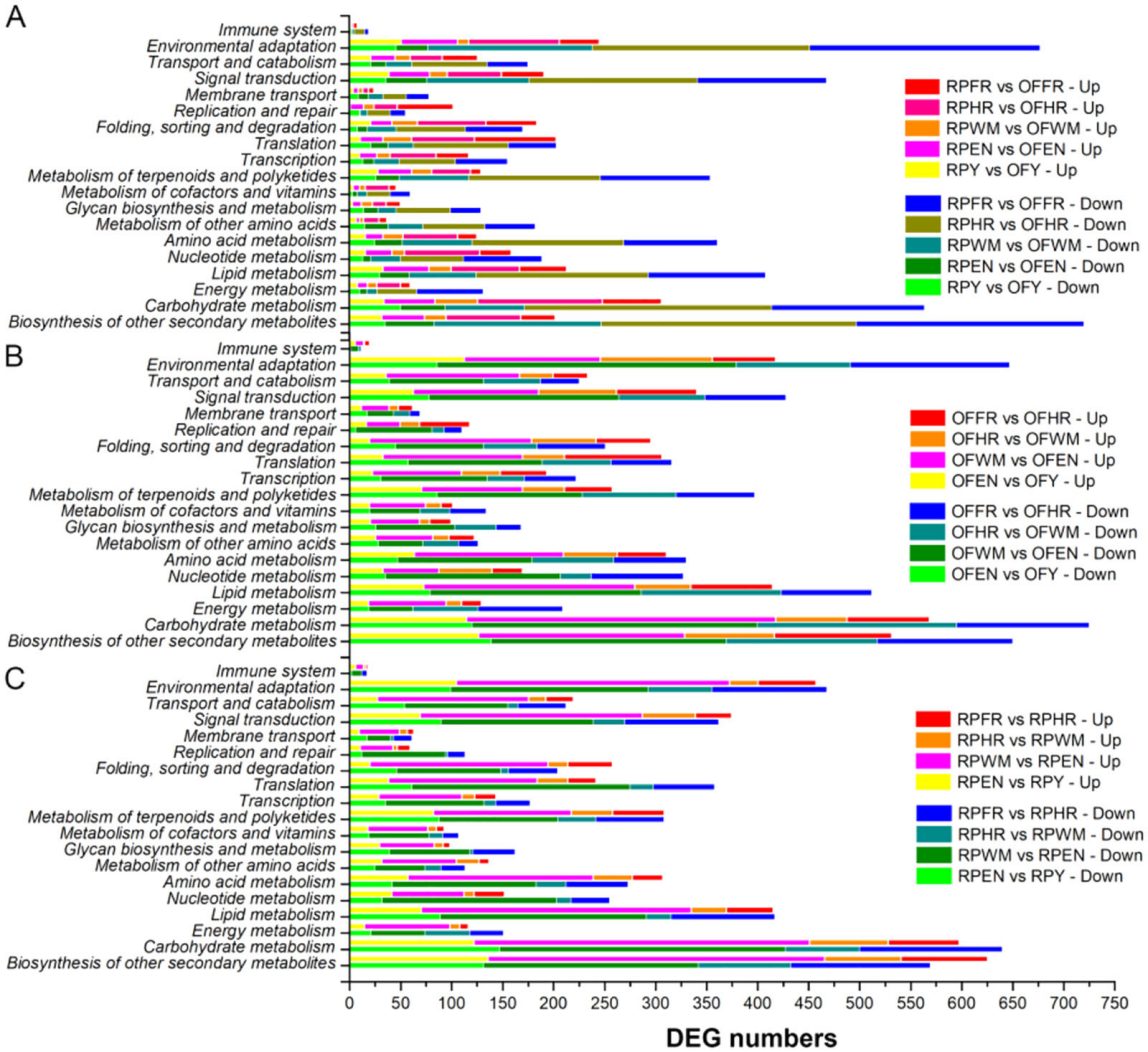
Kyoto Encyclopedia of Genes and Genomes (KEGG) pathway analysis for differentially expressed genes (DEGs). (A) Comparisons between the same developmental stages under rain‐proof (RP) and open‐field (OF) cultivations. (B) Comparisons between the adjacent stages of fruit development under OF cultivation. (C) Comparisons between the adjacent stages of fruit development under RP cultivation.

The functional annotation of DEGs via KEGG enrichment analysis (Figure 6 and Table S5) revealed 125 affected pathways in fruits grown in RP and OF cultivations. The enriched pathways were primarily associated with 10 metabolic pathways (biosynthesis of other secondary metabolites, carbohydrate metabolism, energy metabolism, lipid metabolism, nucleotide metabolism, amino acid metabolism, metabolism of other amino acids, glycan biosynthesis and metabolism, metabolism of cofactors and vitamins, and metabolism of terpenoids and polyketides), 4 genetic information processing pathways (transcription, translation, folding, sorting and degradation and replication and repair), 2 environmental information processing pathways (membrane transport and signal transduction), 1 cellular processes pathway (transport and catabolism), and 2 organismal systems pathways (immune system and environmental adaptation).

In the three comparison groups (Figure 6A–C), the DEGs were mainly enriched in the pathways related to environmental adaptation, signal transduction, transcription, metabolism of terpenoids and polyketides, nucleotide metabolism, amino acid metabolism, lipid metabolism, carbohydrate metabolism, and biosynthesis of other secondary metabolites. In the majority of the seven most enriched pathways, the number of downregulated genes was greater than the number of upregulated genes.

### 3.7 DEGs associated with sugar metabolism in jujube fruit in RP and OF cultivations

**FIGURE 7:**
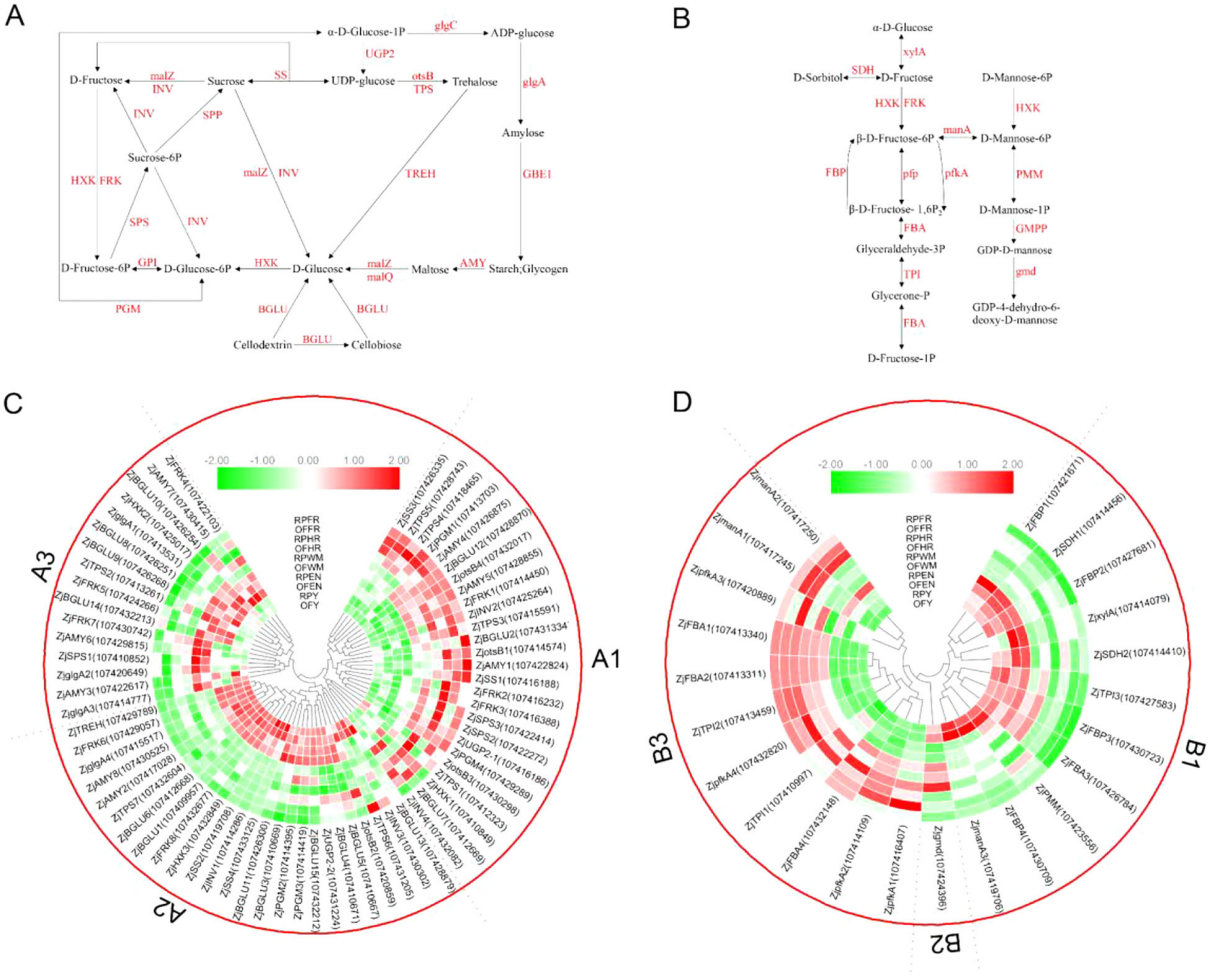
The metabolic map of sugar components and a heatmap of the expression levels of genes associated with sugar metabolism pathways. (A) Starch and sucrose metabolism pathways (map 00500). (B) Fructose and mannose metabolism pathways (map 00051). (C) Heatmap of the expression levels of genes related to the starch and sucrose metabolism pathways. (D) Heatmap of the expression levels of genes related to the fructose and mannose metabolism pathways. The color scale shown at the bottom right corner represents log2‐transformed FPKM counts. FPKM, fragments per kilobase of million mapped.

The pathways associated with sugar metabolism specifically, starch and sucrose metabolism (map 00500) and fructose and mannose metabolism (map 00051) showed significantly enriched DEGs. We reconstructed these complex pathways (Figure 7A,B) and analyzed the gene expression of genes associated with the sugar metabolism pathways using the RNA‐Seq data (Figure 7C,D). Based on the expression profiles of the 90 genes (starch and sucrose metabolism: 4 *Zj*glgA, 8 *Zj*AMY, 15 *Zj*BGLU, 3 *Zj*HXK, 8 *Zj*FRK, 4 *Zj*INV, 3 *Zj*SPS, 4 *Zj*SS, 2 *Zj*UGP2, 4 *Zj*otsB, 7 *Zj*TPS, 1 *Zj*TREH, and 4 *Zj*PGM genes; fructose and mannose metabolism: 1 *Zj*xylA, 2 *Zj*SDH, 4 *Zj*FBP, 4 *Zj*pfkA, 4 *Zj*FBA, 3 *Zj*TPI, 3 *Zj*manA, 1 *Zj*PMM, and 1 *Zj*gmd genes) associated with sugar metabolism, we found that the genes related to starch and sucrose metabolism could be divided into three subgroups, A1–A3 (Figure 7C), and the genes related to fructose and mannose metabolism could be divided into two subgroups, B1–B3 (Figure 7D).

Most genes in groups A1 and B3 displayed high expression levels at the HR and FR stages and had lower expression levels in the other stages. In contrast, the genes in groups A2 and B1 displayed high expression levels at the Y and EN stages, and had lower expression levels in the other stages. In groups A3 and B2, gene expression levels increased during the early stages and decreased in the middle stages of fruit development.

The gene expression patterns in groups A1 and B3 appeared similar to the accumulation pattern of sucrose, whereas those in groups A2 and B1 appeared similar to the accumulation pattern of fructose and glucose.

**FIGURE 8:**
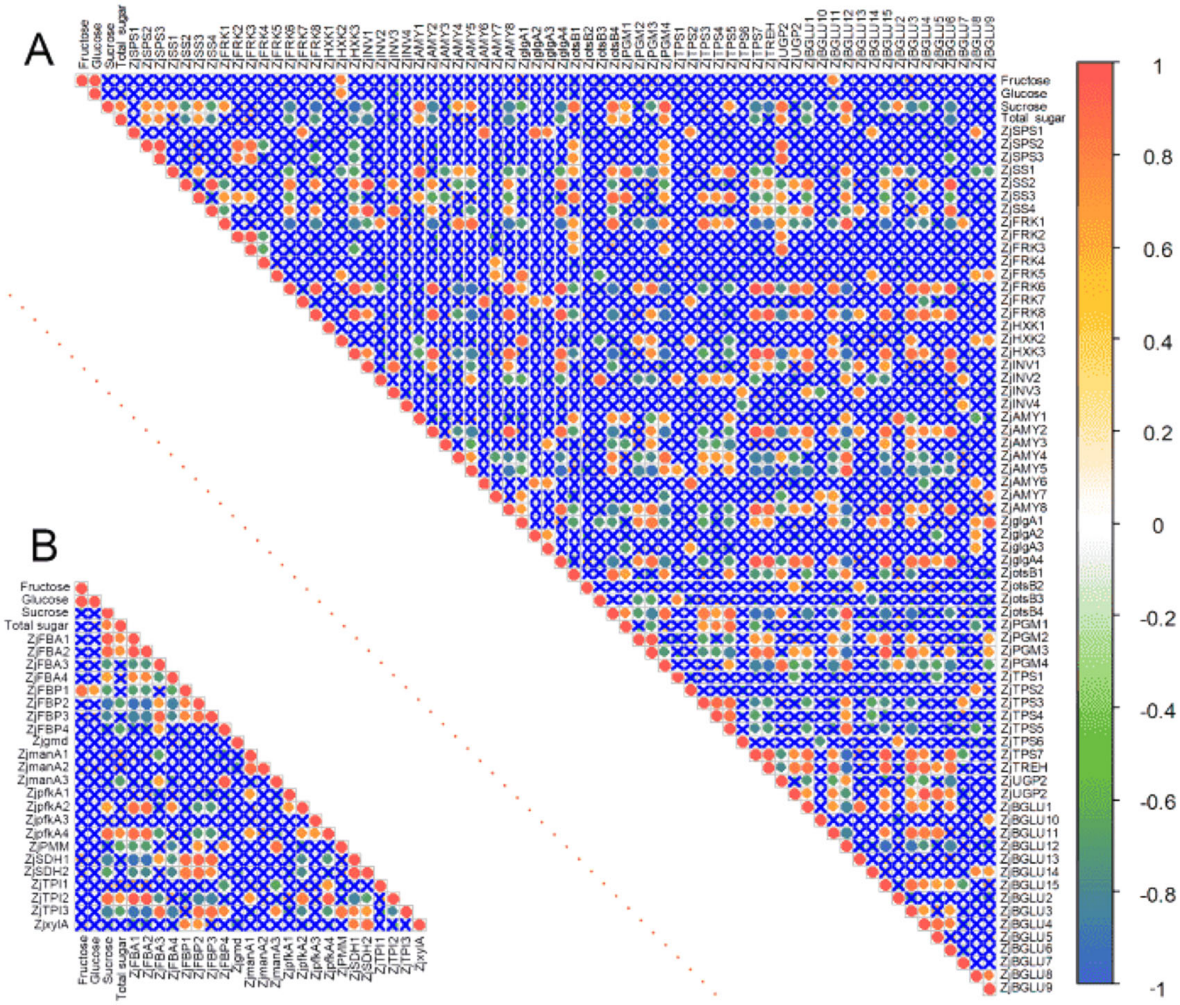
Correlation analysis of sugar levels with the expression levels of genes related to sugar metabolism in jujube fruit. The correlation analysis was performed using the expression levels of the sugar metabolism‐related genes in the original FPKM counts and the sugar contents (fructose, glucose, sucrose, and total sugar) during the five developmental stages of jujube fruit in rainproof and open‐field cultivation. The correlation coefficient without significance at the 0.05 level is marked with the “×” symbol. Red and yellow circles indicate that the correlation is positive and significant at the 0.05 level, and the blue and green circles indicate that the correlation is negative and significant at the 0.05 level. (A) The correlation analysis of sugar contents with the 67 genes under starch and sucrose metabolism (map 00500). (B) The correlation analysis of sugar contents with the 23 genes under fructose and mannose metabolism pathways (map 00051).

We performed a correlation analysis to further explore the associations between the expression levels of these 90 genes and the sugar contents (including fructose, glucose, sucrose, and total sugar content) of fruits (Figure 8). We identified 3 genes (*Zj*HXK2, *Zj*BGLU11 and *Zj*FBP1) that presented significant (<0.05) positive correlations with fructose levels, 2 genes (*Zj*HXK2 and *Zj*FBP1) that presented significant positive correlations with glucose levels, 22 genes (*Zj*SPS2, *Zj*SPS3, *Zj*SS1, *Zj*SS3, *Zj*FRK1, *Zj*AMY1, *Zj*AMY4, *Zj*AMY5, *Zj*otsB1, *Zj*otsB4, *Zj*PGM1, *Zj*PGM4, *Zj*TPS5, *Zj*UGP2, *Zj*BGLU12, *Zj*BGLU2, *Zj*FBA1, *Zj*FBA2, *Zj*FBA4, *Zj*pfkA2, *Zj*pfkA4, and *Zj*TPI2) that presented significant positive correlations with sucrose levels, and 13 genes (*Zj*SPS2, *Zj*SPS3, *Zj*SS3, *Zj*AMY1, *Zj*otsB4, *Zj*PGM1, *Zj*PGM4, *Zj*UGP2, *Zj*BGLU12, *Zj*FBA1, *Zj*FBA2, *Zj*pfkA2, and *Zj*TPI2) that presented significant positive correlations with total sugar levels. Moreover, 25 genes (*Zj*SS2, *Zj*SS4, *Zj*FRK6, *Zj*FRK8, *Zj*HXK3, *Zj*INV1, *Zj*AMY2, *Zj*AMY8, *Zj*glgA1, *Zj*glgA4, *Zj*PGM3, *Zj*TPS7, *Zj*TREH, *Zj*BGLU1, *Zj*BGLU11, *Zj*BGLU15, *Zj*BGLU3, *Zj*BGLU4, *Zj*BGLU6, *Zj*FBA3, *Zj*FBP1, *Zj*FBP2, *Zj*FBP3, *Zj*SDH1, *Zj*SDH2, and *Zj*TPI3) presented significant negative correlations with sucrose levels, and 17 genes (*Zj*SS2, *Zj*SS4, *Zj*FRK6, *Zj*FRK8, *Zj*HXK3, *Zj*INV1, *Zj*AMY2, *Zj*AMY8, *Zj*glgA4, *Zj*TPS7, *Zj*BGLU1, *Zj*BGLU6, *Zj*FBP2, *Zj*FBP4, *Zj*manA3, *Zj*PMM, and *Zj*TPI3) presented significant negative correlations with total sugar levels.

**FIGURE 9:**
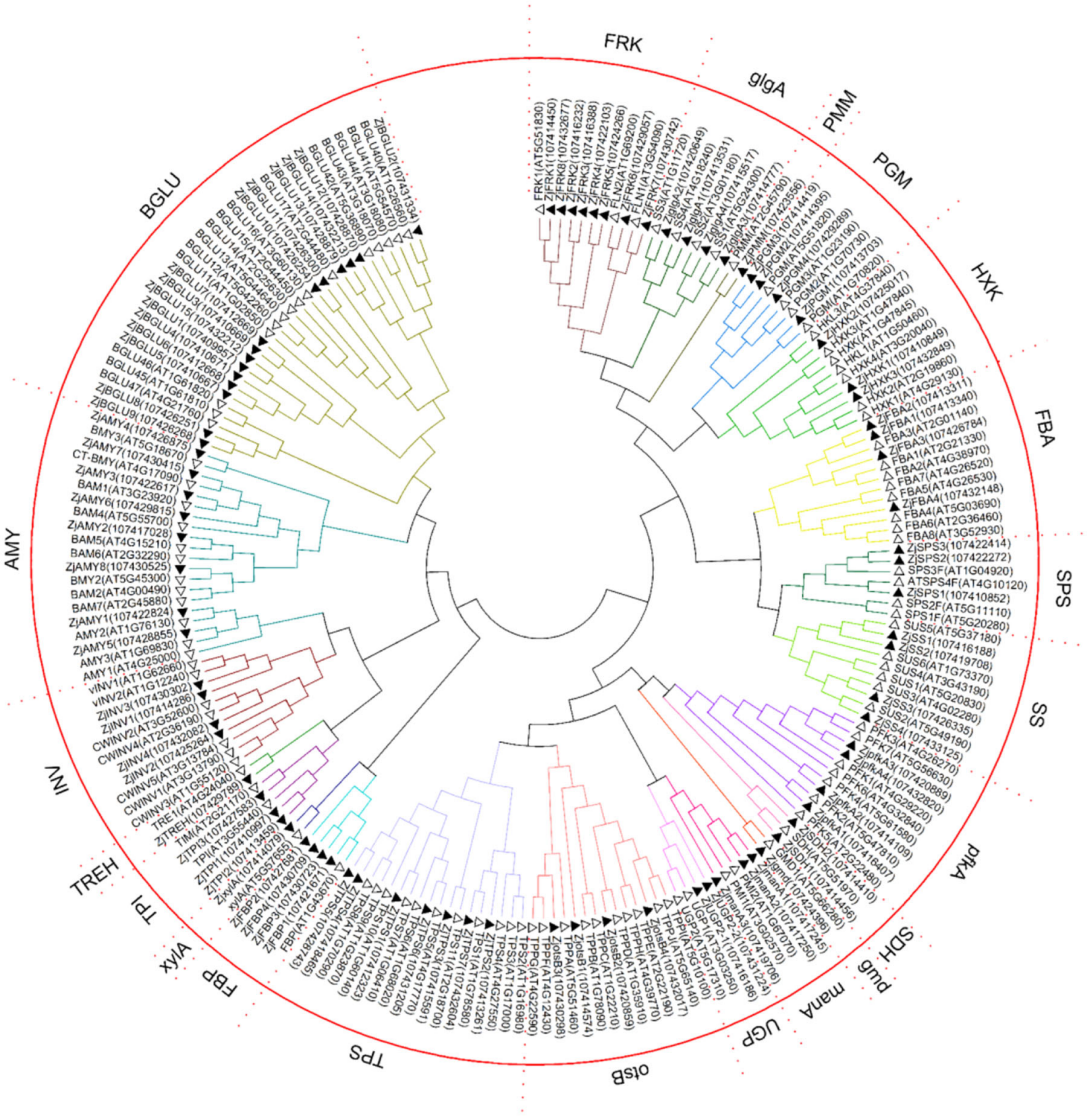
Phylogenetic tree of the genes of jujube and *Arabidopsis*. The small white triangles represent the 90 proteins of jujube, and the small black triangles represent the 110 proteins of *Arabidopsis*.

To further characterize the genes involved in sugar pathways metabolism, we used ClustalX aligned the sequences and constructed a phylogenetic tree with 90 genes of jujube and 110 genes of *Arabidopsis* (Figure 9). The results indicated that the different genes involved in the sugar metabolism pathway in jujube clustered with the corresponding genes in *Arabidopsis*. Additionally, we resolved the systemic relationship of the invertase gene (*Zj*INV) in jujube, The results showed that this gene could be divided into two categories, namely, cell wall invertase (CWINV) (*Zj*INV2 and *Zj*INV4), and vacuolar acid invertase (vINV) (*Zj*INV1 and *Zj*INV3).

### 3.8 Co‐expression network analysis and identification of hub genes related to sugar biosynthesis

A total of 20164 genes were included in the analysis. After data filtering, a total of 15123 genes were used for gene network construction. As shown in Figure S1, there are no outliers in the samples, which ensures that the data can be further analyzed. The one‐step function was used for network construction using parameters as follow: soft threshold power 6, minimum module size 30, merge CutHeight 0.25.

**FIGURE 10:**
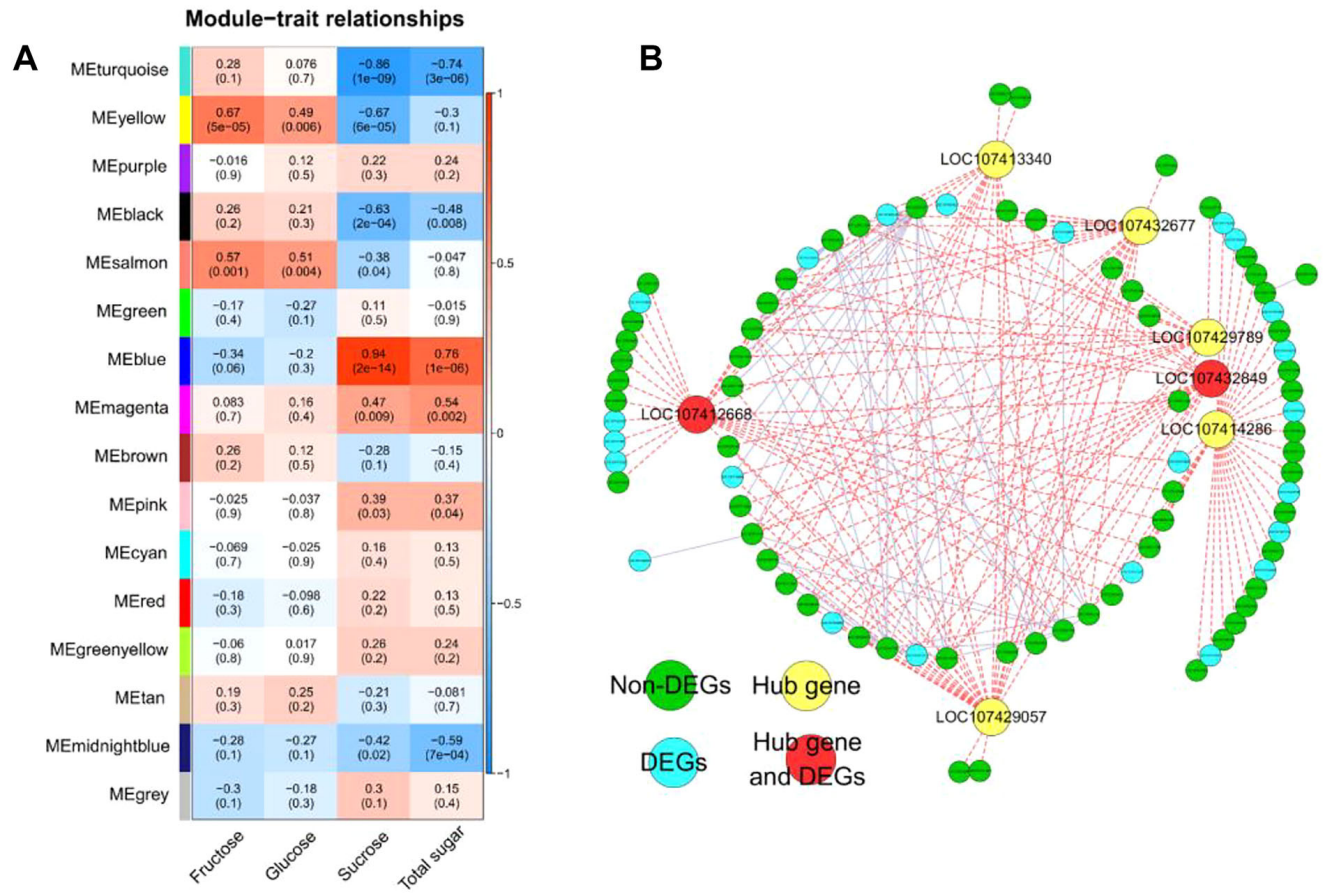
Module‐trait relationships analysis and co‐expression network visualization. (A) Module–trait relationships. Each row represents a module, and the correlation coefficient and the e‐value are shown in each square. Each column corresponds to a sugar composition. (B) Network analysis of the genes related to sugar metabolism. The red dotted line represents the degree of connection between the hub gene and other genes. DEGs, differentially expressed genes.

As shown in Figure 10, 16 modules were identified which are labeled in different colors based on genes expression profiles, of which 7 modules were highly associated with sugar content. The results showed that salmon and yellow module were significantly positively correlated with fructose and glucose content, blue module was significantly positively correlated with sucrose, and turquoise, yellow, and black modules were negatively correlated with sucrose. In addition, blue and magenta modules were significantly positively correlated with total sugar content, while turquoise and midnight blue modules were significantly negatively correlated with total sugar content.

Given the large number of genes in the seven highly correlated modules, the file of network relationships of 90 genes which related to sugar metabolism were extracted from the total gene edge file. The files were sorted by weight value and the first 200 pairs of network connections were used to establish interaction networks. Highly degree (≥15) nodes in expression networks were defined as hub genes, which are often associated with biological processes and interactions.

The 91 genes and 199 correlations edges were shown in the interaction networks showed. Interestingly, 90 genes are all from the turquoise module. It is obvious that a total of seven hub genes were identified, of which two (LOC107412668: *Zj*BGLU6, LOC107432849: *Zj*HXK3) are DEGs.

GO enrichment analysis of the 91 genes was performed. The results showed that 91 genes were significantly enriched in related items such as carbohydrates, cell cycle, and fruit development (Figure S2), which indicated that genes affecting sugar content might also affect fruit development, because sugar, as a signaling molecule and metabolite, was affecting many biological processes (Koch, 1996; Smeekens, 1998).

**FIGURE 11:**
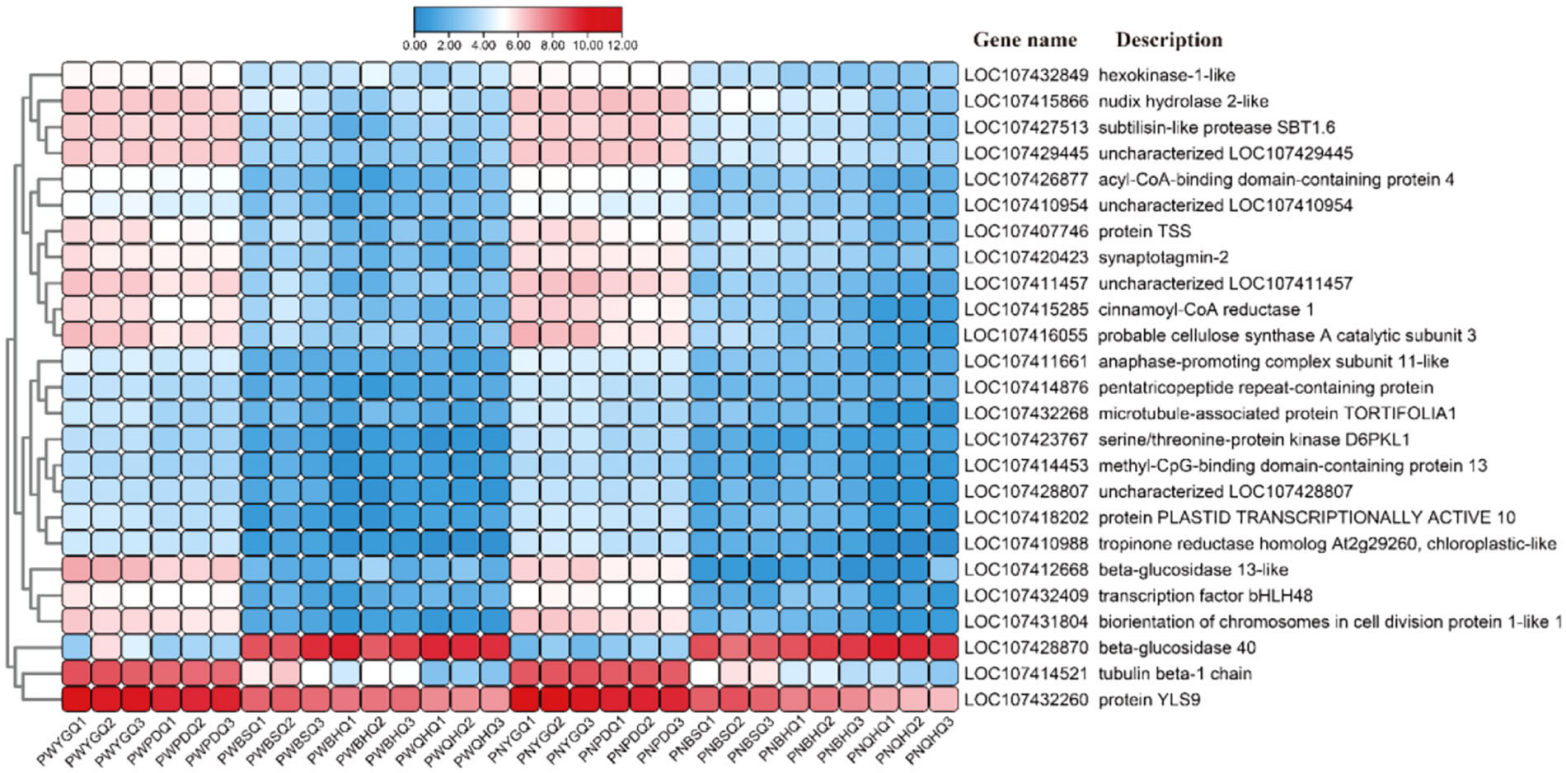
Heatmap of 25 DEGs with jujube fruit developmental under rain‐proof (RP) and open‐field (OF) cultivations.

The interaction network contains 25 differentially expressed genes (Figure 10B), and we use their FPKM values to draw heatmap of the expression. The results showed that the expression of 25 genes showed a trend of increasing first and then decreasing (Figure 11). The expression was higher in the young fruit stage and the expansion stage, and gradually decreased in the later stage of fruit development. It is worth noting that three sugar‐related DEGs (LOC107428870: *Zj*BGLU12, LOC107412668: *Zj*BGLU6, LOC107432849: *Zj*HXK3) have been found which have been identified above section. These results suggest that these genes may play important roles in changes in sugar accumulation in jujube fruit development between under RP and OF cultivations.

### 3.9 Validation of the expression levels of DEGs detected during fruit ripening

**FIGURE 12:**
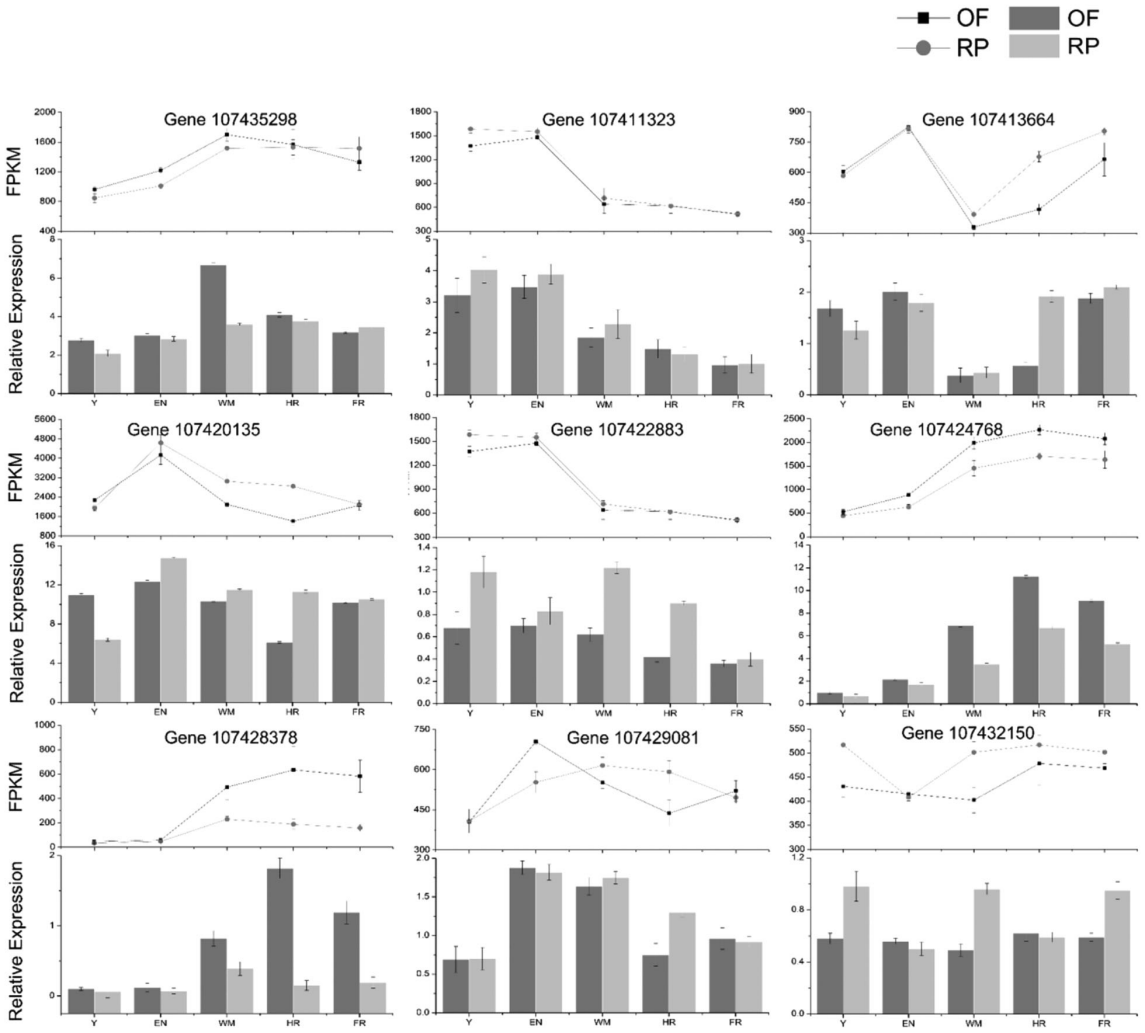
qRT‐PCR verification of the expression levels of 9 genes selected from the transcriptome data. qRT‐PCR, quantitative real‐time polymerase chain reaction.

We performed (qRT‐PCR) using nine genes to verify the reliability of our transcriptome data from the RNA‐Seq analysis of five different developmental stages of jujube fruit (Figure 12). The expression levels of the nine genes were in agreement with the qRT‐PCR results.

## 4 DISCUSSION

### 4.1 The sugar content of jujube fruits changes under RP cultivation

RP cultivation is a simple form of facility agriculture, and has gradually become one of the most popular cultivation techniques for some fruit trees (Li et al., 2009). RP, cultivation can increase the fruit yield (Duan et al., 2019; Xu et al., 2017), reduce disease (Meng et al., 2012), and improve fruit quality (Gao et al., 2016; Meng et al., 2017; Yan‐Nan et al., 2016). The sugar contents of most fruit species grown under RP cultivation are significantly higher than those of fruits grown under OF cultivation (Huang et al., 2009; Tian et al., 2019). However, some studies have also reported the opposite trend (Meng et al., 2017).

In this study, the accumulation patterns of fructose, glucose, sucrose, and total sugar did not change. Glucose and fructose contents were high in the early stages of fruit development and decreased markedly in the later stages. Although the sucrose content could not be detected in the early stages, sucrose was abundant in the later stages. This phenomenon has also been reported in other fruits, such as watermelon (*Citrullus lanatus*) (Yativ et al., 2010). However, the total sugar contents showed an increasing trend throughout the fruit ripening period, and this was in accordance with the results of Song et al. (Song et al., 2019). Sugar of which fructose, glucose, and sucrose are the main is one of the main determinants of jujube fruit flavor. Surprisingly, the sugar levels were remarkable in jujube fruits under RP cultivation than in those under OF cultivation, which made the fruit sweeter. Therefore, the fruit quality of jujube fruits was been greatly improved under RP cultivation.

### 4.2 Transcriptome profiles of jujube fruits grown in RP and OF cultivations

Our results (Figure 3C,D) indicated that in both cultivation modes, the number of DEGs in the EN versus WM comparison was significantly higher than those in all other comparisons between stages. This suggested that the transformation from the EN stage to the WM stage is a key period of nutrient accumulation during fruit inclusion, as well as the most metabolically active period during the development of jujube fruits. The number of DEGs decreased in the later stages, and among these DEGs, the number of downregulated genes was higher than that of upregulated genes. These patterns suggest that jujube fruit maturation is accompanied by metabolic changes that allow the accumulation of some fruit inclusions, such as the jujube stone. Moreover, the expression levels of genes became relatively stable, and fewer genes were involved in metabolism during the later stages of fruit development. The number of DEGs was higher in the later stages of RP‐cultivated fruits than in the later stages of OF‐cultivated fruits, indicating that RP enhanced intrinsic metabolic activities during fruit development. On comparing the developmental stages of RP‐and OF‐cultivated fruits, we found that the number of DEGs was higher in the later developmental stages than in the early stages. This suggested that the influence of environmental conditions on intrinsic accumulation in fruits increases with fruit development and maturation.

Previous studies have shown that sucrose is involved in the regulation of strawberry fruit development and ripening (Jia et al., 2013). The results of this study show that the period of rapid sucrose accumulation was also the period with the largest number of DEGs. Moreover, the rate of sucrose accumulation and the number of DEGs showed similar trends. Therefore, we speculate that since sugar is the most important component of the jujube fruit, sucrose accumulation can also act as a signal that regulates of jujube fruit development and maturity.

Sucrose can not only be used as a substrate for plant growth, but also can induce or suppress the expression of genes involved in other pathways (Koch, 1996). For example, previous studies have shown that higher levels of sugar can inhibit the expression of photosynthetic genes, and that this plays an important role in the feedback inhibition of photosynthesis. When carbohydrates are depleted, plants show positive regulation and enhanced gene expression; in contrast, when carbohydrates are abundant, plants exhibits the opposite trend (Koch, 1996). The of GO (Figure 5) and KEGG enrichment (Figure 6) analyses revealed that among the pathways with the most enriched genes, the number of down‐regulated genes exceeded the number of up‐regulated genes. Therefore, we speculate that in this study, the high levels of sugar accumulation in the later stages of fruit development and RP treatment promoted overall sugar accumulation in the fruit. This acted as a signal to inhibit the expression of genes in other metabolic pathways, thereby regulating the energy balance in the plant.

### 4.3 Expression changes of critical genes involved in sugar metabolism under RP cultivation

Several critical genes encode enzymes including sucrose phosphate synthase (SPS), sucrose synthase (SS), invertase (INV), and hexokinase (HXK)—involved in sugar metabolism in the jujube fruit. Thus, these genes and enzymes regulate the levels of sugar components and the accumulation of sugar (Huang et al., 2016a). Our results show that RP cultivation did not change the pattern of accumulation of sugar components, but increased the levels of sugar components in jujube fruits, likely due to the differential expression of genes. As expected, many DEGs were significantly enriched in pathways related to the biosynthesis/catabolism of glucose, fructose, galactose, and sucrose, including glycolysis/gluconeogenesis, fructose, and mannose metabolism, galactose metabolism, and starch and sucrose metabolism.

SPS and SS are critical rate‐limiting enzymes involved in sucrose metabolism. SPS irreversibly catalyzes the reaction of uridine diphosphate glucose and 6‐phosphate fructose to produce 6‐phosphate sucrose, which is then converted to sucrose under the action of protein phosphatase (PP). SS reversibly catalyzes the synthesis or degradation of sucrose during plant growth. In this study, we identified two genes of *Zj*SPS (*Zj*SPS2 and *Zj*SPS3) in jujube fruit, and found that two genes of *Zj*SS (*Zj*SS1 and *ZJ*SS3) were up‐regulated during jujube fruit development under RP cultivation. This suggests that the genes encoding SS and SPS were more highly expressed under RP cultivation than under OF cultivation. Moreover, the total gene expression of all *Zj*SS genes was higher than that of *Zj*SPS genes during the entire developmental. Thus, we speculate that the contribution of *Zj*SS to sugar accumulation in jujube fruits is greater than that of *Zj*SPS.

Invertase (INV) is known to have a specific effect on the accumulation of sugar content in fruits (Husain et al., 2001; Yang et al., 2013). cINV is thought to mainly regulate the unloading of sucrose from the phloem, whereas the vINV in the vacuole is thought to regulate the storage of sucrose and hexose (fructose and glucose) (Bachelier et al. 1997). Moreover, the activity of acid invertase is much lower in high‐sugar watermelon than in low‐sugar watermelon (Yativ et al., 2010). Manipulating the acid invertase gene on chromosome 3 of tomato resulted in low invertase activity, which in turn caused a change in the sugar components, increased the hexose level, and lowered the sucrose level (Husain et al., 2001). In this study, the vINV gene of jujube (*Zj*INV1) (107414286) was highly expressed in the Y and EN stages of fruit development, and its expression levels were decreased or negligible after the EN stage. This is contrary to the sucrose accumulation patterns in our study, and the expression levels of *Zj*INV1 show a significant negative correlation with sucrose levels. We speculate that a high expression level of *Zj*INV1 may inhibit sucrose accumulation in jujube fruits, and is conducive to the synthesis of fructose and glucose.

HXK is an important enzyme in glycolysis and plays an important role in sugar accumulation. Previous studies on grapes have reported that HXK activity is higher before the fruit ripens and that hexose is mainly used for substance metabolism. When the fruit ripens, glycolysis is weakened, and the remaining hexose is mainly used for accumulation (Robinson et al. 1997). Overexpression of an *Arabidopsis thaliana* gene (*At*HXK1) in tomato (*Solanum lycopersicum* L.) causes HXK to be maintained a high level, which is not conducive to the accumulation of fruit sugars (Dai et al., 1999). Our phylogenetic reconstruction showed that *Zj*HXK3 (107432849) was clustered with the *At*HXK1 gene of *Arabidopsis* (Figure 9). The *Zj*HXK3 gene was highly expressed in the early stages of jujube fruit development, and its expression levels decreased in the later stages under rainproof cultivation. Moreover, the expression of *Zj*HXK3 showed significant negative correlations with the levels of sucrose and total sugar. Through WGCNA analysis, it is found that *Zj*HXK3 is the hub gene of the network. Therefore, we speculate that the *Zj*HXK3 gene of jujube has a similar function as the *At*HXK1 gene of *Arabidopsis*, and that high expression levels of *Zj*HXK3 were not conducive to sugar accumulation in jujube fruits.

### 4.4 Effects of ecological factors on the accumulation of sugar content in jujube fruits

The accumulation of sugar content in fruits is regulated by the expression of enzymes related to sugar metabolism. The enzymes related to sugar metabolism have certain ecological adaptability, and their expression is affected by related ecological factors. Temperature and rainfall during fruit growth had an important effect on the sugar content of the fruit, and a moderately high temperature contributed to the accumulation of sugar (Chen et al., 2012). Studies on melon fruit showed that with the increase of treatment temperature, the sugar content and the activities of SPS and SS increased (Ren et al., 2010). Low‐temperature treatment of postharvest kiwi fruit inhibited the increase of SPS activity and the decrease of AI invertase in the fruit. The increase of SPS activity and the decrease of invertase showed a significant positive correlation with the accumulation of sucrose in kiwi fruit (Zhang et al., 2004). When fruit uptakes a large amount of water during the fruit growing season, the sugar of the fruit is diluted, while moderate water stress will increase the sugar content of the fruit (Kobashi et al., 2000). The results of the study on citrus showed that from the EN stage to the fruit maturity, the soil moisture content had a great influence on the fruit quality, and the soil was slightly drier, which was more conducive to increasing the sugar content of the juice (Lez‐Altozano & Castel 1999). Under moderate water stress, the SS activity of orange fruit was enhanced, which promoted the accumulation of photosynthetic products (Hockema & Etxeberria, 2001). Therefore, RP cultivation increased the average temperature of air and soil in the whole fruit development cycle, avoided the scouring of fruits by rainfall, and reduced soil humidity; we speculate the changes of these ecological factors increased the gene expression of *Zj*SPS and *Zj*SS in the fruit of jujube, inhibited the gene expression of *Zj*INV, and promoted the accumulation of sugar content in jujube fruits; similar results were found in peaches (*Prunus persica* L.) and strawberries (*Fragaria × ananass*a Duch.) (Kobashi et al., 2000; Wang et al., 2000).

## 5 CONCLUSION

This is the first transcriptomic study to elucidate the molecular mechanisms underlying changes in sugar levels in Chinese jujube plants grown under RP cultivation. The sugar content of jujube fruits was significantly higher under RP cultivation than under OF cultivation, although the sugar compositions, and total sugar accumulation patterns were comparable between these groups. Comparative transcriptomic analyses suggested that the genes encoding SPS, SS, INV, and HXK were differentially expressed during jujube fruit development, RP changed the microclimate of fruit growth environment, mainly including temperature, humidity, and water conditions, and then regulated the expression of these key genes, were responsible for the increase in sugar content in Chinese jujube. We speculate that in jujube fruits, sucrose may be a signal molecule that regulates fruit development and maturity. Our results provide genetic resources for the study of sugar accumulation and metabolism in Chinese jujube, and this study provides a reference for the study of molecular mechanisms underlying trait changes in other tree species under RP cultivation.

## AUTHOR CONTRIBUTIONS

**Qing Ji**: data curation; formal analysis; funding acquisition; investigation; writing–original draft; writing–review and editing. **Ruifang Wang**: writing–original draft; writing–review and editing. **Kai Chen**: supervision; writing–review and editing. **Zhengwan Xie**: investigation. **Sunyang Li**: formal analysis; writing–review and editing. **Dawei Wang**: formal analysis; investigation. **Ao Zhang**: writing–review and editing. **Yumei Xu**: writing–review and editing. **Shenghui Li**: writing–review and editing. **Junjun Cui**: writing–review and editing. **Sha Liu**: writing–review and editing. **Jun Zhou**: resources; supervision; writing–original draft; writing–review and editing. **Lihu Wang**: data curation; formal analysis; funding acquisition; writing–original draft; writing–review and editing.

## CONFLICT OF INTEREST STATEMENT

The authors declare no conflicts of interest.

## Supporting information

Supplementary Materials: Table S1: Specific primer pairs.

Table S2: Statistic Reads of RNA‐Seq data.

Table S3: Gene expression results of samples.

Table S4: The DEGs between different comparison groups.

Table S5: KEGG pathway enrichment analysis of DEGs.
Figure S1: Sample clustering to detect outliers. Figure S2 The most enriched GO Terms of 91 genes.
